# Systemic nimodipine affects pericyte calcium signaling, resting hemodynamics and neurovascular coupling in healthy mouse brain

**DOI:** 10.1016/j.neurot.2025.e00614

**Published:** 2025-05-21

**Authors:** Jessica Meza-Resillas, Finnegan O'Hara, Syed Kaushik, Michael J. Stobart, Noushin Ahmadpour, Meher Kantroo, John Del Rosario, Megan C. Rodriguez, Dmytro Koval, Chaim Glück, Bruno Weber, Jillian L. Stobart

**Affiliations:** a College of Pharmacy, University of Manitoba, Winnipeg, Manitoba, Canada; b Centre on Aging, University of Manitoba, Winnipeg, Manitoba, Canada; c Institute of Pharmacology and Toxicology, University of Zurich, Zurich, Switzerland

**Keywords:** Nimodipine, Pericyte, Cerebral blood flow, Two-photon microscopy

## Abstract

Nimodipine is a L-type voltage gated calcium channel blocker commonly given to patients after a sub-arachnoid hemorrhage. It is known to dilate cerebral arteries and affect brain pericytes that express voltage gated calcium channels. Here, we systemically administered nimodipine (1 ​mg/kg; i.p.) and measured brain pericyte calcium transients and single-vessel hemodynamics in the brains of mice by two-photon microscopy. We find that nimodipine reduces calcium transients in all types of pericytes, from ensheathing to thin-strand cells, at different locations in the vascular network. This induces local vasodilation of vessels closer to penetrating arterioles but decreases blood cell velocity. These vascular consequences are due to systemic nimodipine effects because direct brain application of nimodipine caused blood cell velocity to increase. Nimodipine treatment also reduced further dilation during neurovascular coupling throughout the vascular network. Overall, this suggests that nimodipine can change cerebrovascular hemodynamics by altering pericyte physiology and these are important considerations for the clinical use of this drug.

## Introduction

Subarachnoid hemorrhage (SAH) is caused by bleeding between the arachnoid layer and pia matter, commonly from a ruptured aneurysm [1,2]. Patients who survive the initial bleeding event are at a risk of developing delayed cerebral ischemia, which often correlates with vasospasm of cerebral arteries visible by angiography and reduced cerebral blood flow (CBF) [3]. The only treatment approved by the FDA for recovery from SAH is nimodipine, an L-type voltage gated calcium channel (VGCC) blocker [1]. Nimodipine is typically administered in the days following SAH, and it is more hydrophobic than other dihydropyridines, allowing it to readily cross the blood-brain-barrier [4]. In SAH, nimodipine is known to improve mortality and patient outcomes; however, the precise mechanism of how nimodipine benefits the chronic phase after SAH is unclear. Evidence suggests nimodipine alleviates arteriole vasospasms (as seen in an SAH animal model [5]), but in most patient studies, it fails to increase CBF [1,[6], [7], [8], [9]]. This is theorized to occur because healthy blood vessels may dilate in response to nimodipine and “steal” blood flow from vessels constricted by SAH, leading to no overall change in total CBF [1]. Therefore, it is important to determine how systemic nimodipine affects blood flow throughout the healthy cerebrovascular network to understand how it may dilate mural cells on different blood vessels.

The cerebrovascular network is organized into different microvascular zones based on the branch order and population of mural cells that are located within the basolateral membrane of the blood vessels. Specifically, the pial and penetrating arterioles are wrapped by vascular smooth muscle cells [10,11] that are most prone to vasospasms during SAH [5]. The arteriole-capillary transition zone (ACT) is situated within the 1st to 4th branches after the penetrating arteriole and contains ensheathing pericytes [11]. Ensheathing pericytes express alpha-smooth muscle actin (α-SMA) like vascular smooth muscle cells [11,12] and they contract blood vessels in the ACT with rapid dynamics [[13], [14], [15], [16]]. Cellular contraction is induced by elevated intracellular Ca^2+^ in these pericytes and a drop in Ca^2+^ is associated with quick dilation during neurovascular coupling (NVC) [[17], [18], [19]]. This process is critical to ensure an adequate blood supply reaches active brain areas. Deeper in the vascular network, fine capillaries five or more branches from the penetrating arteriole contain distal, thin-strand capillary pericytes [11]. Thin-strand pericytes are named for their long, narrow processes that extend along capillaries [11,12]. These pericytes have low α-SMA expression that is only detectable with specific fixation procedures [20], but nevertheless, they can constrict capillaries [21], albeit with slower kinetics [22]. This suggests they maintain capillary tone and resistance and may play a role in NVC and local blood supply in healthy brain. While ensheathing and thin-strand pericytes have often been confused in the past due to their similarities, it is now clear that they are distinct populations because they reside in specific brain microvascular zones [11,12], they have unique morphologies [11,12], they have discrete transcriptomic and proteomic profiles [10,23,24], and they have different functional roles in regulating CBF [11,12,25].

Both ensheathing pericytes and thin-strand pericytes respond to dihydropyridines in animal tissue slices or cultured cells [13,16,17,[26], [27], [28]]. Nimodipine or nifedipine decreases fluctuations in ensheathing pericyte intracellular Ca^2+^ that is linked to cellular contraction [13,16,27,28]. In distal thin-strand pericytes, the activity of VGCC appears to be less robust than in ensheathing pericytes, since VGCC blockers mildly reduce or do not affect spontaneous Ca^2+^ signaling in thin-strand pericytes in brain slices [17,27], and these channels are not activated during pressure-induced capillary constriction unlike upstream ensheathing pericytes and smooth muscle cells [16]. However, VGCC are reportedly expressed by thin-strand pericytes [10,29], and the contribution of these channels to thin-strand pericyte Ca^2+^ signaling and cortical hemodynamics *in vivo* remains unknown.

Here, we systemically administered nimodipine to healthy mice to investigate how this common clinical drug affects pericyte Ca^2+^ signaling *in vivo* and hemodynamics throughout the cerebrovascular network during vasomotion and NVC. Our results suggest that nimodipine may have systemic effects on CBF regulated by pericytes in healthy blood vessels, which has important implications for blood distribution throughout the vascular network in health and disease.

## Materials and Methods

### Mouse lines

Two transgenic mouse lines were used to observe brain pericytes: 1) Acta2-RCaMP1.07 (JAX: 028345) mice that express RCaMP1.07, a genetically encoded Ca^2+^ indicator, under the Acta-2 (α-SMA) promoter and 2) *Pdgfrb*-CreERT2 (JAX 029684 or 030201) crossed to GCaMP6s^fl/fl^ (JAX 028866) mice, which express GCaMP6s (genetically encoded Ca^2+^ indicator) in mural cells after tamoxifen activates CreERT2. Tamoxifen was dissolved in corn oil (25 ​mg/ml) and given once per day for 5 consecutive days by oral gavage (200 ​μl per dose). GCaMP6s was visible 3 weeks later. The Animal Care Committee at the University of Manitoba approved all procedures in accordance with the Canadian Council on Animal Care and we report the details based on the ARRIVE 2.0 guidelines [30]. Animals of both sexes were used for experiments and data was collected between 3 and 12 months old.

### Cranial window surgery

Mice were prepared for two-photon imaging in two separate surgical procedures as described previously [31,32]. First, while the mouse was anesthetized with isoflurane (4 ​% induction, 2 ​% maintenance) an incision was made down the midline and an aluminum head post was fixed at the back of the head with light-cured dental acrylic (Tetric EVOflow). The left hemisphere of the skull was not covered with dental acrylic. During the second surgery two to three days later, the mouse was anesthetized with midazolam, medetomidine, and fentanyl (0.5, 5, and 0.05 ​mg/kg) and a dental drill was used to remove a portion of the skull over the left hemisphere. A 3 ​mm ​× ​3 ​mm piece of sapphire glass was sealed with dental acrylic over the surface of the brain to form the chronic cranial window. Animals received meloxicam (2 ​mg/kg s.c. every 24 ​h) and slow release buprenorphine (0.5 ​mg/kg s.c. every 72 ​h) for six days after the first surgery.

### Intrinsic optical imaging

A map of the whisker barrel somatosensory cortex was prepared by intrinsic optical imaging at least 2 weeks after cranial window surgery. The imaging setup contained 630 ​nm red light LEDs and a Basler Ace camera (acA2040-55um) with 1.0× SilverTL telecentric lens (Edmund Optics). Animals with a cranial window were anesthetized (isoflurane; 4 ​% induction, 1.5 ​% maintenance) and placed under the camera and LEDs. Images of the cortical surface were acquired while individual whiskers were vibrated at 90 ​Hz for 5 ​s. The vibration induced local changes in barrel blood flow, which were detected as increased absorption of red light within the images brought on by the change in oxygenated hemoglobin. After 10–20 trials, a clear map of the area corresponding to each whisker was evident (see example map in Fig. 8A). Pericytes of interest were identified within these mapped regions during the two-photon microscopy as described below.

### Two-photon microscopy during systemic nimodipine

Animals recovered for 3 weeks after cranial window surgery. Following ketamine/xylazine (90/10 ​mg/kg, i.p.) anesthesia, fluorescent dextran (30 ​μl of 2.5 ​% w/v fluorescein or Texas Red; 70,000 ​MW in saline) was injected via a catheter into the tail vein as described previously [33]. Through the cranial window, fluorescence images of pericytes and blood plasma were collected on a Bruker two-photon microscope (Ultima) with 20× objective (Olympus XLUMPLFLN, 1.0 NA), galvo scanners, and Ti:sapphire laser (Coherent Chameleon Ultra) set at 990 ​nm (fluorescein/RCaMP) or 940 ​nm (GCaMP/Texas Red). In the first imaging session, fields of view with brain pericytes at specific locations (i.e. branch order) in the vascular network were chosen for future re-localization during the second nimodipine imaging session. Each animal underwent two 2-photon imaging sessions: 1) pre-nimodipine baseline session where no drug was administered and 2) post-nimodipine i.p. injection (1 ​mg/kg in PEG-400) where imaging started at least 20 ​min after the injection. These two imaging sessions were at least 3 days apart. Movies from each field of view were collected (128 ​× ​128 pixels, 11–15 frames per second) for 60 ​s to acquire pericyte Ca^2+^ events. Two-photon line scans were collected by directing the two-photon laser in a one-dimensional path perpendicular and parallel to the vessel to generate kymographs that captured the diameter (μm) and position of blood cells (BC) in the plasma so that BC velocity (mm/second) and BC flux (cells/second) could be approximated. Line scans were collected at a rate of ∼1.8–4 ​ms per scan, depending on the length of the line drawn across the vessels. For consistency across imaging sessions, we applied the same imaging parameters (i.e. laser power, zoom, PMT settings, etc.) and scanning pattern for the line scan path. To stimulate the whisker barrel cortex for neurovascular coupling, we applied a weak electric stimulus (500 ​μA ​at 4 ​Hz; applied with STG4008 stimulus generator for 5 ​s) with electrodes within the whisker pad during the imaging trials.

### Acute nimodipine application and imaging

Mice were prepared with chronic cranial windows and fields of view with pericytes were selected for two-photon imaging as described above. Under isoflurane (4 ​% induction, 2 ​% maintenance) the cranial window was removed by penetrating the dental acrylic with a dental drill. Sham solution (1 ​% PEG400) or nimodipine (10 ​μM in 1 ​% PEG400) was applied to the exposed brain for 20 ​min, while fluorescent dextran was injected into the tail vein. After drug application of the surface, a 2 ​% agarose gel and a new glass coverslip were applied. During two-photon imaging, the anesthesia was switched from isoflurane to ketamine/xylazine (90/10 ​mg/kg) and data was acquired from the fields of view as described above.

### Blood pressure measurements

Blood pressure and body temperature were measured with a CODA Monitor non-invasive tail cuff blood pressure system [34,35]. Anesthetized mice (ketamine/xylazine) were placed on a heating pad with temperature control monitored by a rectal thermometer. The tail was inserted into the occlusion cuff and volume pressure recording system. Blood pressure readings were taken at 15-s intervals over 5-min periods, for a total of 10 measurements.

### Immunohistochemistry

Mice were transcardially perfused with 20 ​mL of ice-cold oxygenated artificial cerebrospinal fluid *(*125 ​mM NaCl, 2.5 ​mM KCl, 2.5 ​mM CaCl_2_, 2 ​mM MgCl_2_, 26 ​mM NaHCO_3_, 1.25 ​mM NaH_2_PO_4_, 25 ​mM glucose; pH 7.4) at ∼25 ​ml/min followed by 60 ​ml of ice-cold 2 ​% paraformaldehyde (Millipore-Sigma; P6148). Brains were post-fixed in ice-cold 4 ​% paraformaldehyde for 3 ​hrs followed by cryoprotection at 4 ​°C overnight in 30 ​% sucrose. After tissue was frozen on dry-ice, 300 ​ ​μm sections were cut on a cryostat (Leica). Sections were washed with TBST (0.05 ​% Triton X-100; three times for 10 ​min) and PBST (1 ​% Triton X-100; once for 10 ​min), and incubated for 48 ​h in 5 ​% Normal Donkey Serum (NDS) ​+ ​1 ​% PBST ​+ ​primary antibodies at 4 ​°C. Washes were repeated in 1 ​% PBST (5 times for 30 ​min each) and secondary antibodies in1% PBST ​+ ​1 ​% NDS were applied for 48 ​h at 4 ​°C. Following incubation, sections were again rinsed 3 times with 1 ​% PBST and 2 times with PBS. Slices were then dehydrated with increasing methanol concentrations: 25 ​%, 50 ​%, 75 ​%, 100 ​% for 30 ​min per step and cleared by incubation in benzyl benzoate: benzyl alcohol (2:1) for 3 hrs. Images of cleared sections were collected with a Zeiss LSM 810 confocal microscope at 40×. The following antibodies were used: primary antibodies-rabbit-anti-Cav1.2 (1:200, Alomone labs, ACC-003), rat-anti-CD13 (1:250, Bio-Rad; MCA2183EL), and goat anti-CD31 (1:200, R&D systems, AF3628); secondary antibodies-donkey-anti-rabbit-IgG-AlexaFluor488 (1:1000, Invitrogen), donkey-anti-rat-IgG-AlexaFluor405 (1:1000, Invitrogen), and donkey-anti-goat-IgG-AlexaFluor647 (1:1000, Invitrogen).

### Brain pericyte Ca^2+^ image processing

In ImageJ, pericyte subcellular compartments (soma and processes) were manually selected and these regions of interest (ROIs) were imported into MATLAB. Data was processed in MATLAB using the CHIPS toolbox (https://github.com/EIN-lab/CHIPS) [36]. First, signal overlap between the green and red channel was reduced by spectral unmixing with a linear unmixing matrix applied to all data. Second, signal intensities from manual ROIs (soma/process) were extracted and the change in fluorescence (dF/F_o_) was calculated with the first 5 s of the trials as the baseline F_o_. Traces of dF/F_o_ were smoothed by band-pass filters and the findpeaks function was applied to identify Ca^2+^ transients and their amplitude. Third, an algorithm (originally from Ellefsen et al. [37]) was used to automatically detect the active regions of Ca^2+^ events (in three dimensions (x, y, and time) that exceeded three times the standard deviation of the baseline. Automatically detected Ca^2+^ events were confirmed to occur within the vessel structure, and the frequency (signals/minute) was calculated as the total number of Ca^2+^ events that occurred within the manually selected regions of interest. To analyze the frequency components of our Ca^2+^ traces, we computed the discrete Fourier transform of the trace vector using MATLAB's Fast Fourier Transform function (fft). We then derived the single-sided amplitude spectrum and power spectral density (PSD) of the signal. The most common frequency peak was identified within the range of 0.01 ​Hz–0.5 ​Hz, which was used for the power spectral density representation. The frequency corresponding to the maximum amplitude in the power spectral density was extracted for each calcium trace, and these values were used for statistical analysis to compare pre-nimodipine and post-nimodipine groups. We also determined the basal RCaMP or GCaMP fluorescence intensity (F_o_), which was the average fluorescence of the first 2.5 s from the calcium movies, to compare the effects of pre and post-nimodipine treatment on intracellular calcium levels.

### Blood vessel hemodynamic image processing

The CHIPS toolbox in MATLAB was used to process the hemodynamic line scans. Diameter was extracted from the kymographs by applying a full-width at half maximum calculation. From the trace of vessel diameter, the fluctuations over time were used to approximate the vasomotion frequency (vessel wall oscillations/minute) and amplitude of vasomotion (vasomotor index; ΔD/D; change in diameter/mean diameter). Blood cell velocity and flux were calculated by applying a radon transformation algorithm to the line scans that ran parallel to the vessel wall. Occasionally, single deviant values that exceeded known data ranges for diameter, velocity, and flux were detected in the traces of hemodynamic data. These values were replaced with the average of the two nearest data points. For velocity and flux data, we ran the Rosner's test (minimum ​= ​2 and maximum ​= ​15 outliers) to identify and remove outliers that were potentially introduced as an artifact from the radon transformation, particularly for very fast flowing vessels. Final data traces were interpolated to the same time scale and smoothed at 10 data points per second before plotting.

### Statistical analysis

Statistics were processed in RStudio 2023.09.0 ​+ ​463. The data was confirmed to be normally distributed with Shapiro-Wilk Test and visually by Quantile-Quantile (Q-Q) plots, histograms, and box plots. Data was converted to log normal for statistically comparisons if not normally distributed. Statistical comparisons were done with Linear Mixed Models (LMM) to account for the repeated measures of individual animals and individual pericytes or blood vessels (i.e. random effects in the models) while pharmacology (with or without nimodipine) was the fixed effect. The LMM of best fit with or without the fixed effect was determined by likelihood-ratio testing and Anova test (for their omnibus significance). Pair-wise P values were calculated by Holm-sequential Bonferroni correction. Data was represented with boxplots of the median and quartile values. All means, standard deviations, and P values are presented in the Supplementary Tables. Data and materials are available upon request (Dr. Jillian Stobart; jillian.stobart@umanitoba.ca).

## Results

### Brain pericytes express L-type VGCC subunit Cav1.2

**Fig. 1:**
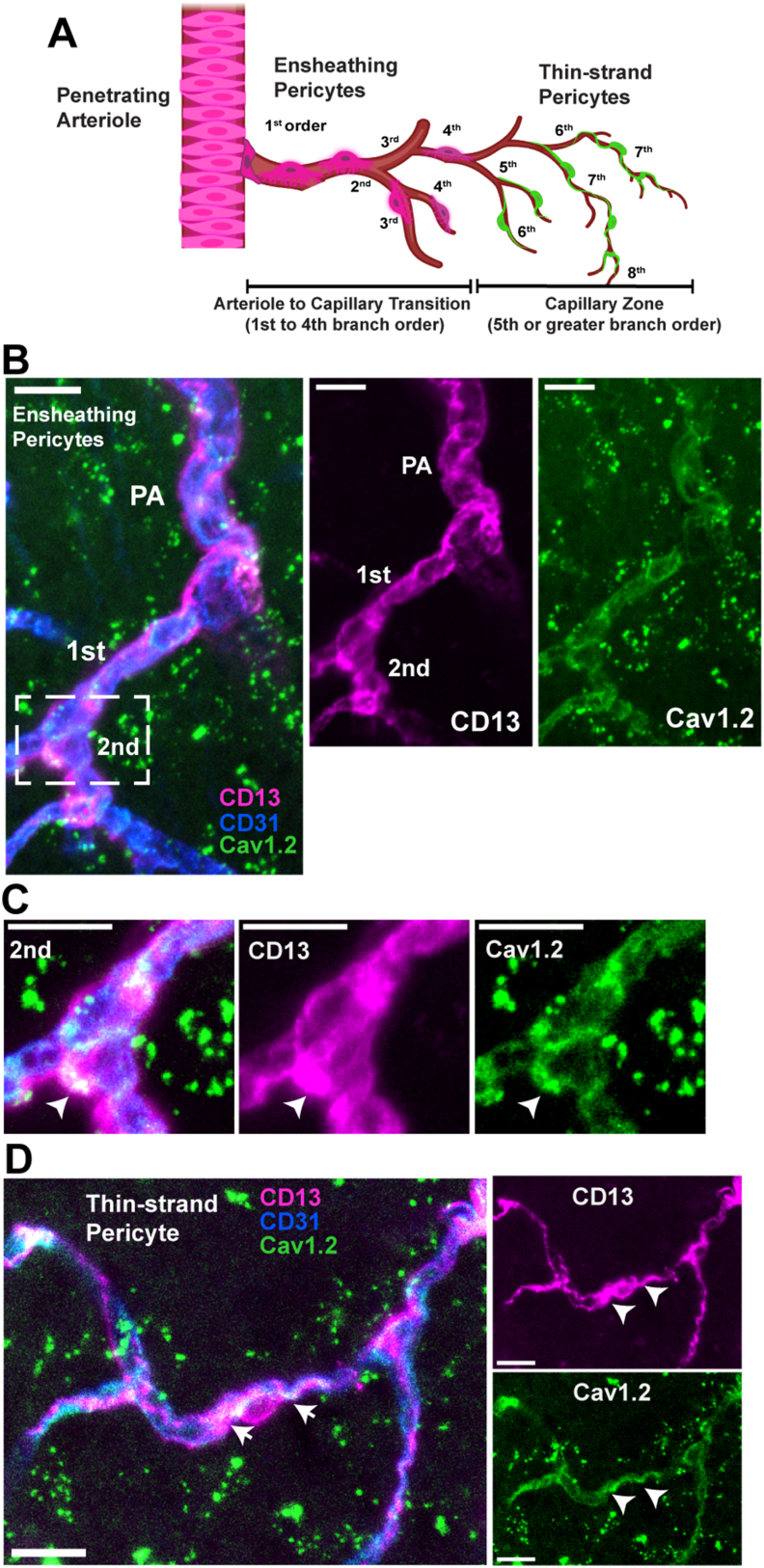
**Brain pericytes express L-type VGCC subunit, Cav1.2.** A) Diagram of the pericyte types and their location within the cerebrovascular network. **B)** Immunohistochemistry shows the antibody for Cav1.2, a subunit of L-type VGCC (green), localized to mural cells (anti-CD13, magenta) near endothelial cells (anti-CD31, blue) in branches of the ACT. **C)** Ensheathing pericytes from a second order vessel indicated in the dashed box in B. Arrowhead indicates the pericyte soma. **D)** Cav1.2 staining (magenta) also localizes to thin-strand pericytes (anti-CD13, green) found more than 5 branches from the penetrating arteriole. Arrowheads indicate the processes. All scale bars ​= ​10 ​μm.

Pericytes reportedly express L-type VGCC subunit, Cav1.2 [10,28,29], but the type of pericyte and location of staining within the cerebrovascular network has not been described. We used cleared, thick mouse brain sections for Cav1.2 immunostaining, and classified ensheathing pericytes as the mural cells located in the ACT (1st to 4th branches after the penetrating arteriole) and thin-strand pericytes as mural cells with narrow processes 5 or more branches from the penetrating arteriole (Fig. 1A), as described above [11]. Cav1.2 staining localized to blood vessels and overlapped with CD13 mural cell marker in both ensheathing pericytes in the ACT (Fig. 1B and C) and thin-strand pericytes on deeper capillaries (Fig. 1C). This Cav1.2 antibody has been knockout validated and provided a true signal compared to no-primary antibody controls (Supplementary Fig. 1), verifying that brain pericytes express the VGCC blocked by nimodipine.

### Systemic nimodipine reduces ensheathing pericyte Ca^2+^ and resting blood vessel hemodynamics in the arteriole-capillary transition zone

**Fig. 2:**
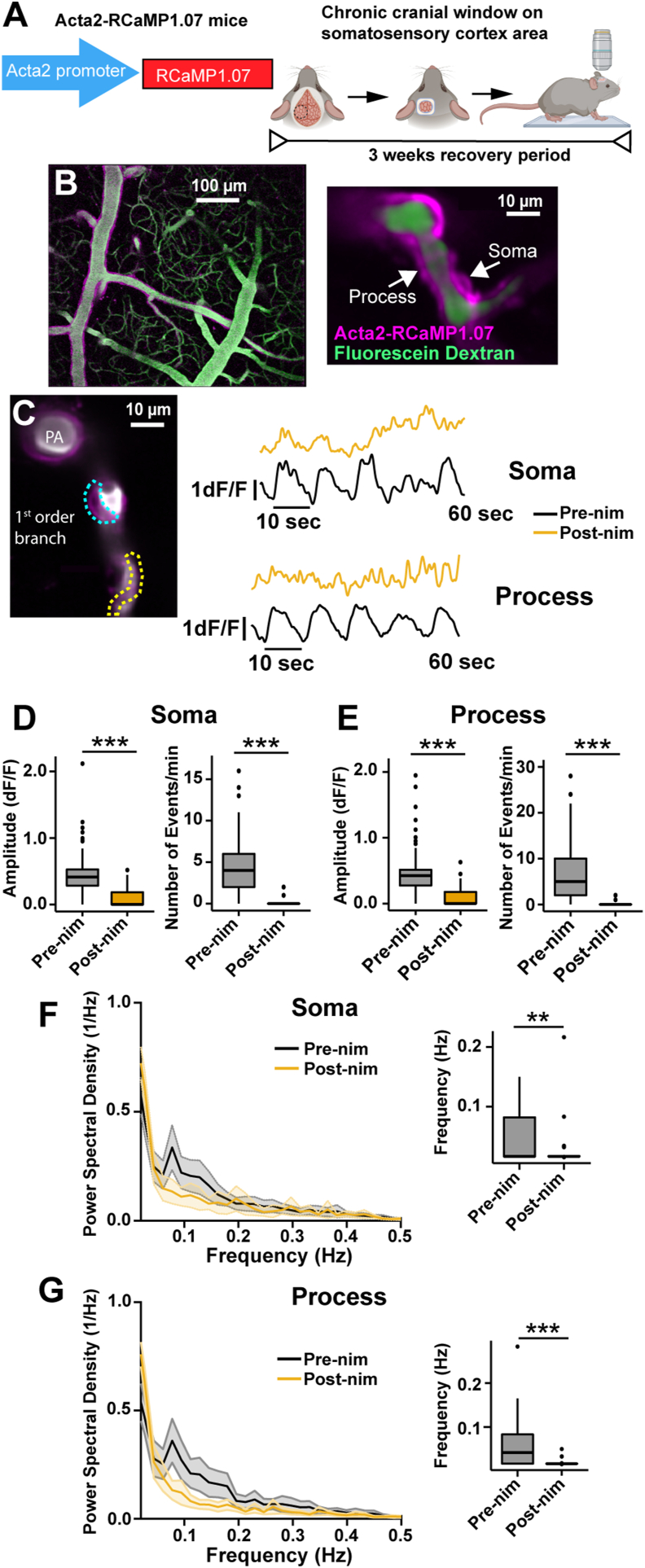
**Nimodipine affects ensheathing pericyte calcium signaling in the ACT. A)***Acta2*-RCaMP1.07 mouse model and chronic cranial window surgical scheme. **B)** Visualization of cerebrovascular network and ACT mural cells (left) expressing RCaMP1.07 (magenta) under αSMA promoter which labeled ensheathing pericytes (right). The blood plasma was labeled with fluorescein dextran (i.v.; green). **C)** Left: Two-photon image of ensheathing pericyte morphological structures- Soma: cyan dashed line; Process; yellow dashed line. Right: Individual Ca^2+^ signaling traces of soma and process from cell on the left. Spontaneous Ca^2+^ signaling properties (amplitude and number of events per min) from ensheathing pericyte somata **D)** and processes **E)** are reduced by nimodipine. Box plots and Power Spectral Density plots of the frequency (Hz) of ensheathing pericyte calcium events from somata **F)** and processes **G)**. n ​= ​43 pericytes from 7 mice. ∗∗∗P ​< ​0.001. Pre-nim ​= ​pre-nimodipine; Post-nim ​= ​post-nimodipine. Means, standard deviations, and exact P values for data from all figures are provided in the Supplementary Tables.

To study the effects of systemic nimodipine on healthy ensheathing pericytes *in vivo*, we utilized *Acta2*-RCaMP1.07 mice which express the red genetically encoded Ca^2+^ indicator RCaMP1.07 under the Acta2 promoter for α-SMA expression (Fig. 2A). By two-photon microscopy through a chronic cranial window, we observed RCaMP expression localized to vascular smooth muscle cells on penetrating arterioles and ensheathing pericytes in the ACT in ketamine/xylazine anesthetized mice (Fig. 2B). We recorded the basal RCaMP fluorescence intensity (Supplementary Fig. 2A) and also fluctuations in fluorescence reflecting changes in intracellular Ca^2+^ in ensheathing pericyte somata and processes in the first imaging sessions (pre-nimodipine) and the same cellular structures were imaged again following systemic nimodipine administration several days later (post-nimodipine; 1 ​mg/kg, i.p.; Fig. 2C). Nimodipine reduced the amplitude and number of Ca^2+^ events in ensheathing pericyte somata and processes (Fig. 2D and E) and decreased the basal fluorescence intensity (F_o_) of each cellular compartment (Supplementary Fig. 2A). Since ensheathing pericyte Ca^2+^ is closely linked to their contractility [[17], [18], [19]] and vascular dynamics are known to have intrinsic ultra-low frequencies associated with properties such as vasomotion [38,39], we calculated the power spectral density of the Ca^2+^ traces by Fast Fourier Transformation. Under pre-nimodipine conditions, the peak frequency of Ca^2+^ in the soma and processes of ensheathing pericytes ranged from 0.05 to 0.1 ​Hz. However, after nimodipine treatment, this frequency pattern for Ca^2+^ was no longer evident, and the most common frequency (i.e. the maximum amplitude from the power spectral density) decreased (Fig. 2F and G).

**Fig. 3:**
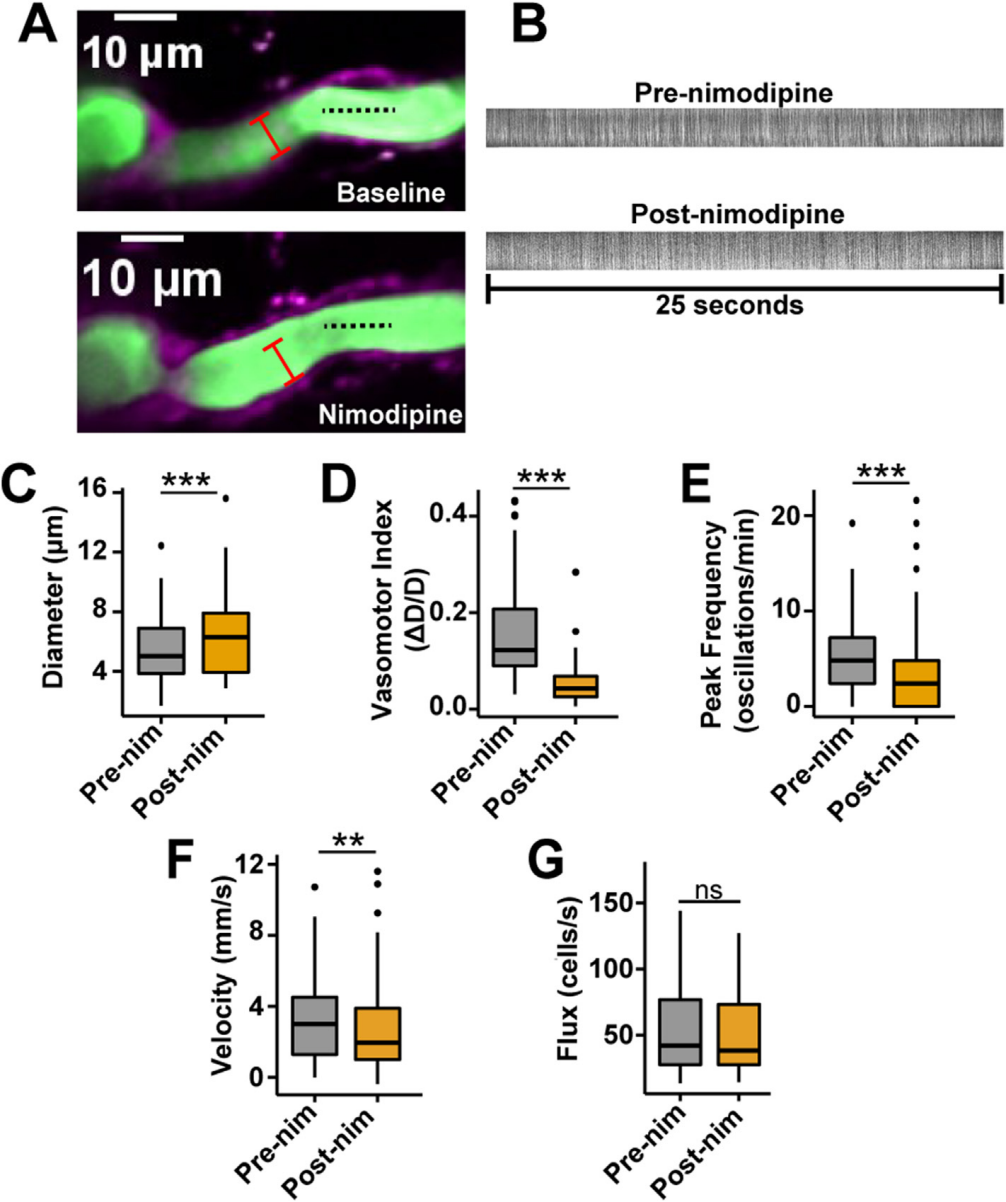
**Nimodipine affects blood flow in the ACT. A)** Example of a 1st order blood vessel (green) covered by ensheathing pericytes (magenta) and the diameter (red line) during baseline and nimodipine. The black dotted line is an example path for a velocity line scan. **B)** Example line scans to measure BC velocity and flux from a 2nd branch order blood vessel. The black spaces are the BCs and the white spaces are blood plasma. Duration of the kymographs ​= ​25 ​s. **C)** Diameter**, D)** vasomotor index**,** and **E)** vasomotion peak frequency of all blood vessels from the ACT covered by ensheathing pericytes are affected by nimodipine. n ​= ​101 blood vessels from N ​= ​7 mice. **F)** BC velocity and **G)** flux of blood vessels from the ACT covered by ensheathing pericytes. n ​= ​101 blood vessels from 7 mice. ∗∗P ​< ​0.01, ∗∗∗P ​< ​0.001. Pre-nim ​= ​pre-nimodipine; Post-nim ​= ​post-nimodipine.

In the blood vessels adjacent to the ensheathing pericytes, we conducted two-photon line scans for vessel diameter, blood cell (BC) velocity and flux based on the green fluorescein fluorescence in the blood plasma (from i.v. injection, 2.5 ​% w/v; Fig. 3A and B). Vessels in the ACT are known to have vasomotion [11,17], which are spontaneous, rhythmic contraction and dilation events that are important for blood delivery into the downstream capillary network [40]. We estimated vasomotor activity by calculating the number of vessel diameter oscillations per minute (vasomotor frequency) and the diameter amplitude change (ΔD/D) which we termed the vasomotor index. Nimodipine increased the mean diameter of all vessels covered by ensheathing pericytes (Fig. 3C) and decreased both vasomotor amplitude and frequency (Fig. 3D and E). Interestingly, systemic nimodipine decreased the mean BC velocity through vessels of the ACT despite the increased vessel diameter (Fig. 3F). However, nimodipine did not alter the mean BC flux overall (Fig. 3G), suggesting that perfusion through the ACT was unaffected.

### Systemic nimodipine has stronger effects on the first branches of the arteriole-capillary transition zone

**Fig. 4:**
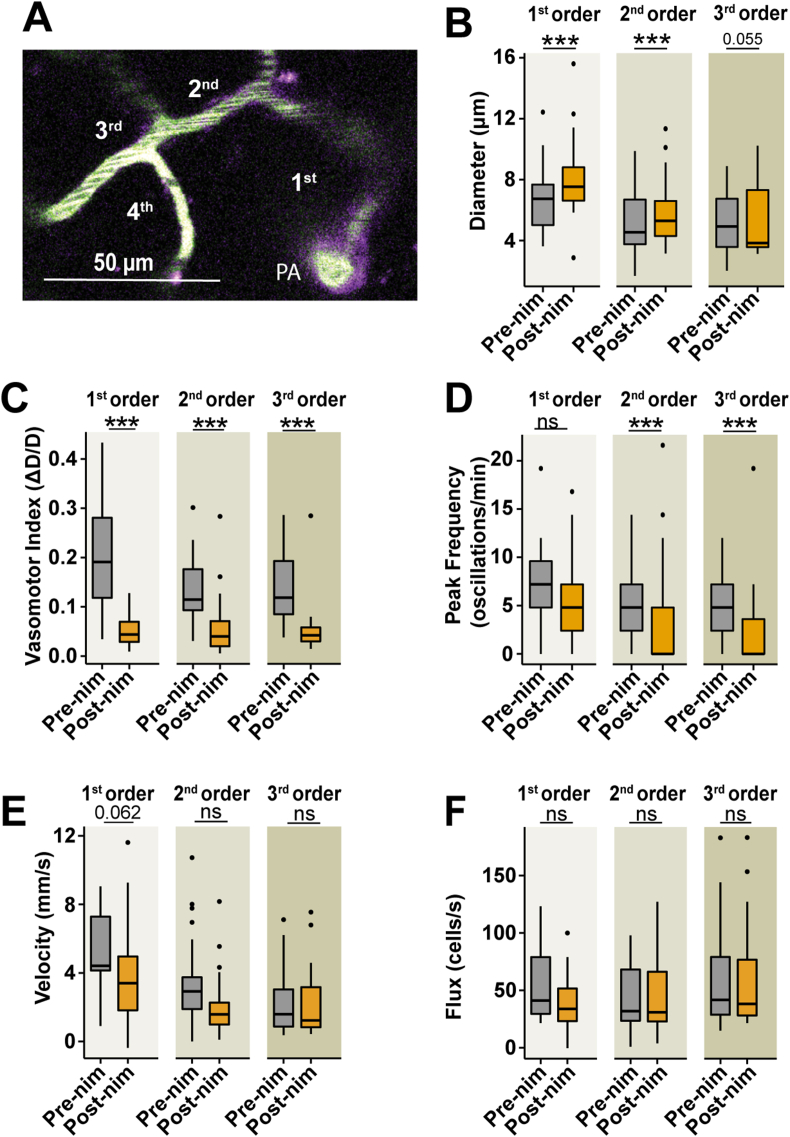
**Nimodipine affects the hemodynamics of specific branches within the arteriole transition zone differently. A)** An example of the branch order classification within the ACT; ensheathing pericytes (magenta), blood vessels (green). PA ​= ​penetrating arteriole. Effects of nimodipine on diameter **(B)*,*** vasomotor index **(C)**, vasomotion peak frequency **(D)**, BC velocity **(E)** and BC flux **(F)** of 1st (b1), 2nd (b2), and 3rd (b3), branch order blood vessels. b1 ​= ​27; b2 ​= ​32; b3 ​= ​29 blood vessels from 7 mice. ∗∗∗P ​< ​0.001. Pre-nim ​= ​pre-nimodipine; Post-nim ​= ​post-nimodipine.

The first branches of the ACT have faster and larger dilation kinetics during NVC, suggesting they have greater basal contractility [16,18,19,22], and possibly stronger susceptibility to L-type VGCC blockade. When considering the effects of nimodipine on blood flow through specific branches (1st to 3rd) of the ACT (Fig. 4A), the 1st and 2nd branches dilated during drug treatment but there was no effect on the mean diameter of the 3rd branch (Fig. 4B). Nimodipine decreased the vasomotor index in all three branches (Fig. 4C) and the vasomotor frequency in the 2nd and 3rd branches (Fig. 4D). Lastly, nimodipine tended to decrease the velocity in the 1st branch (P ​= ​0.062) but did not affect velocity in the 2nd and 3rd branches (Fig. 4E), nor the flux in any branch (Fig. 4F). Overall, these results suggest that the ACT may have a decreasing gradient of VGCC activity since nimodipine had less significant effects as the branch order approached the capillary network. This has implications for the clinical effects of nimodipine.

### Systemic nimodipine affects thin-strand capillary pericytes

**Fig. 5:**
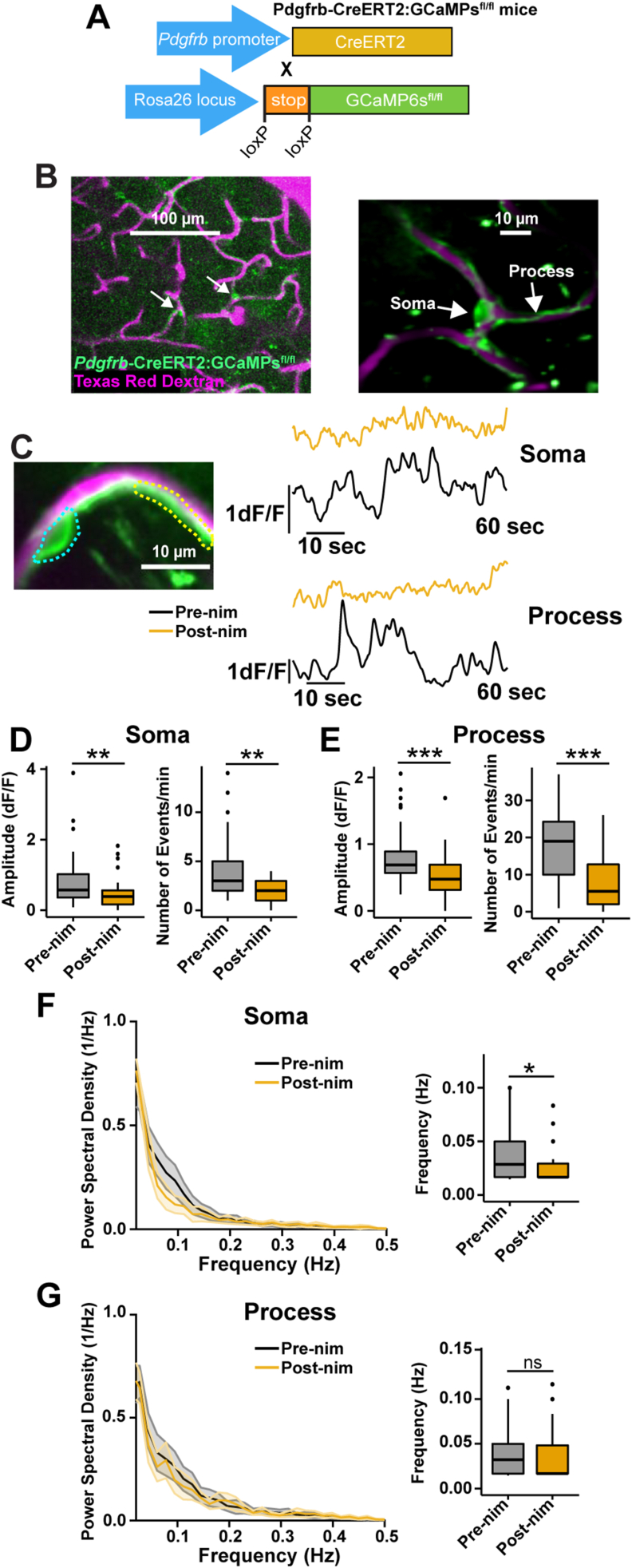
**Nimodipine affects thin-strand pericyte calcium signaling in the capillary network. A)***Pdgfrb*-CreERT2:GCaMP6s^fl/fl^ mouse model. **B)** Thin-strand pericytes expressing Cre-dependent GCaMP6s (green, white arrows) more than 5 branches from the penetrating arteriole and the vasculature (i.v. Texas red dextran injection; magenta). **C)** Left: Two-photon image of thin-strand pericyte morphological structures- Soma: cyan dashed line; Process; yellow dashed line. Right: Individual Ca^2+^ signaling traces of soma and process from left in pre-nimodipine and post-nimodipine conditions. Spontaneous Ca^2+^ signaling properties (amplitude and number of Ca^2+^ events per minute) of thin-strand pericytes somata **D)** and process **E)** are reduced by nimodipine. Box plots and Power Spectral Density (PSD) plots of the frequency (Hz) of ensheathing pericyte calcium events somata **F)** and process **G)** altered by nimodipine. n ​= ​44 pericytes from 7 mice. ∗∗P ​< ​0.01, ∗∗∗P ​< ​0.001. Pre-nim ​= ​pre-nimodipine; Post-nim ​= ​post-nimodipine.

To study thin-strand pericytes deeper in the brain capillary network *in vivo*, we utilized *Pdgfrb*-CreERT2:GCaMP6s^fl/fl^ mice which express inducible Cre recombinase (CreERT2) in all mural cells with platelet-derived growth factor receptor beta (*Pdgfrb;* Fig. 5A). CreERT2 activity was induced by tamoxifen administration and caused the expression of the green genetically encoded Ca^2+^ indicator, GCaMP6s (Fig. 5B), in pericytes including thin-strand pericytes located more than 5 branches from the penetrating arteriole. Similar to ensheathing pericytes (Fig. 2, Fig. 3), systemic nimodipine administration decreased the amplitude and number of Ca^2+^ events in both the somata and processes of thin-strand pericytes (Fig. 5C–E). However, it did not reduce the basal GCaMP fluorescence intensity (F_o_, Supplementary Fig. 2B), suggesting that the resting intracellular Ca^2+^ concentration was unaffected. Thin-strand pericytes are known to have asynchronous spontaneous Ca^2+^ activity that does not correlate with vascular properties like vasomotion [17,18,29], and this was apparent when considering the power spectral density of Ca^2+^ frequencies following Fast Fourier Transformation, as there was a lack of a peak near 0.1 ​Hz (Fig. 5F and G). Following nimodipine treatment, there was a decrease in the frequencies corresponding to the maximum amplitude of calcium events in thin-strand pericyte somata (Fig. 5F), but the frequencies in thin-strand pericyte processes were unchanged (Fig. 5G). Overall, this suggests that nimodipine reduces Ca^2+^ activity in thin-strand pericytes, but only impacts the periodicity of Ca^2+^ events in the somata.

**Fig. 6:**
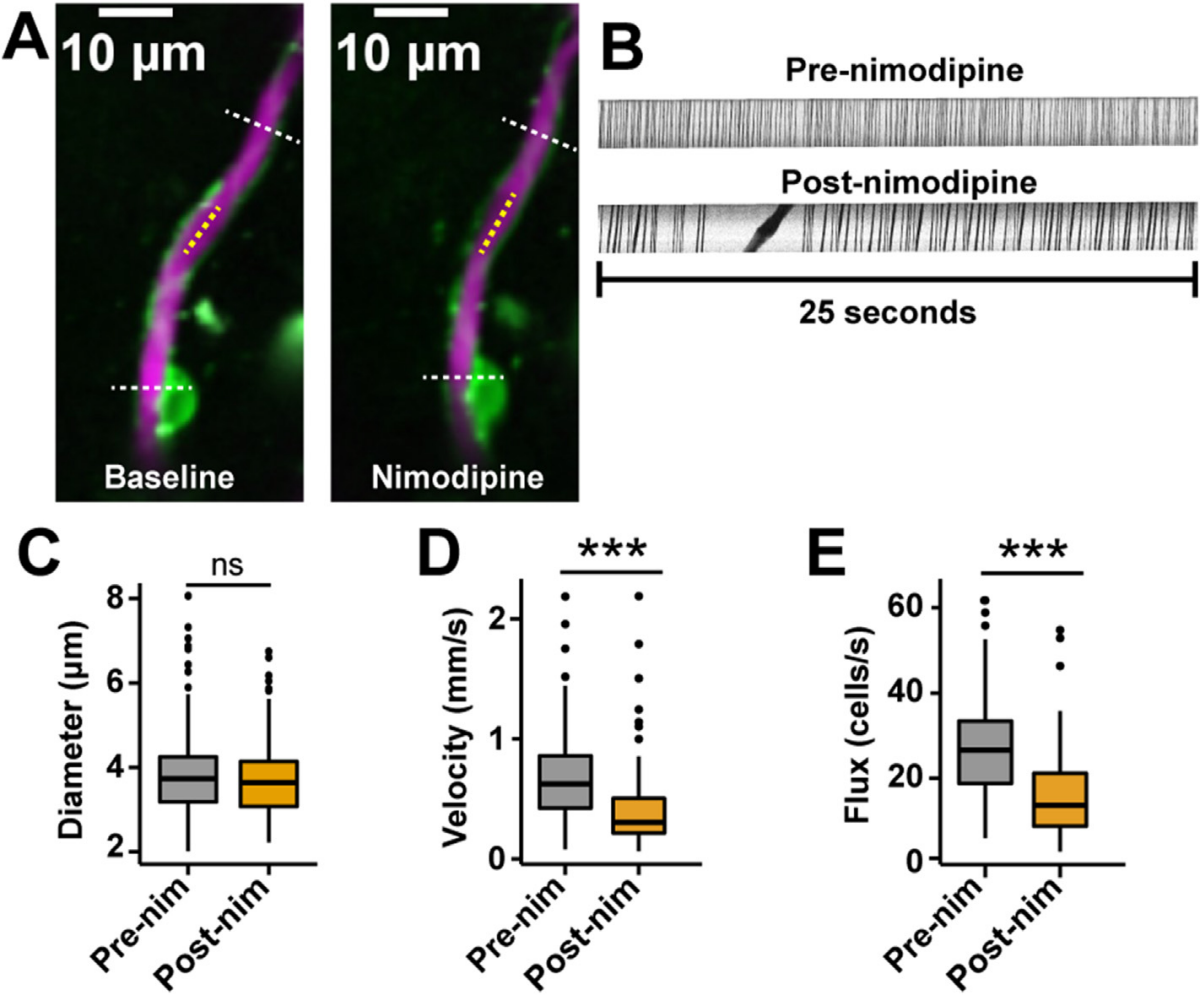
**Nimodipine affects blood cell velocity and flux in the capillaries. A)** Example of a brain capillary (magenta) covered by a thin-strand pericyte (green) and its diameter (white dashed line) in response to nimodipine. The yellow dotted line is an example path for a velocity line scan. **B)** Individual representative kymographs of one brain capillary. The black spaces represent BCs, and the white spaces represent blood plasma. A brief stalling of BCs is apparent in the kymograph post-nimodipine. Duration of the kymographs ​= ​25 ​s. Box plots of diameter **(C),** velocity (**D**) and flux (**E**) of brain capillaries covered by thin-strand pericytes. n ​= ​109 vessels from 7 mice. ∗∗∗P ​< ​0.001. Pre-nim ​= ​pre-nimodipine; Post-nim ​= ​post-nimodipine.

We also measured single-vessel hemodynamics by line scans near thin-strand pericytes following Texas Red dextran fluorescence (2.5 ​% w/v, i.v., Fig. 6A and B). Despite a drop in Ca^2+^ activity, nimodipine had no dilatory effects on capillaries (Fig. 6C). However, nimodipine decreased velocity and BC flux in capillaries (Fig. 6D and E), and caused heterogenous fluctuations in BC velocity because of brief stalling of blood flow (Fig. 6B).

### Acute nimodipine application has different effects on hemodynamics than systemic nimodipine

**Fig. 7:**
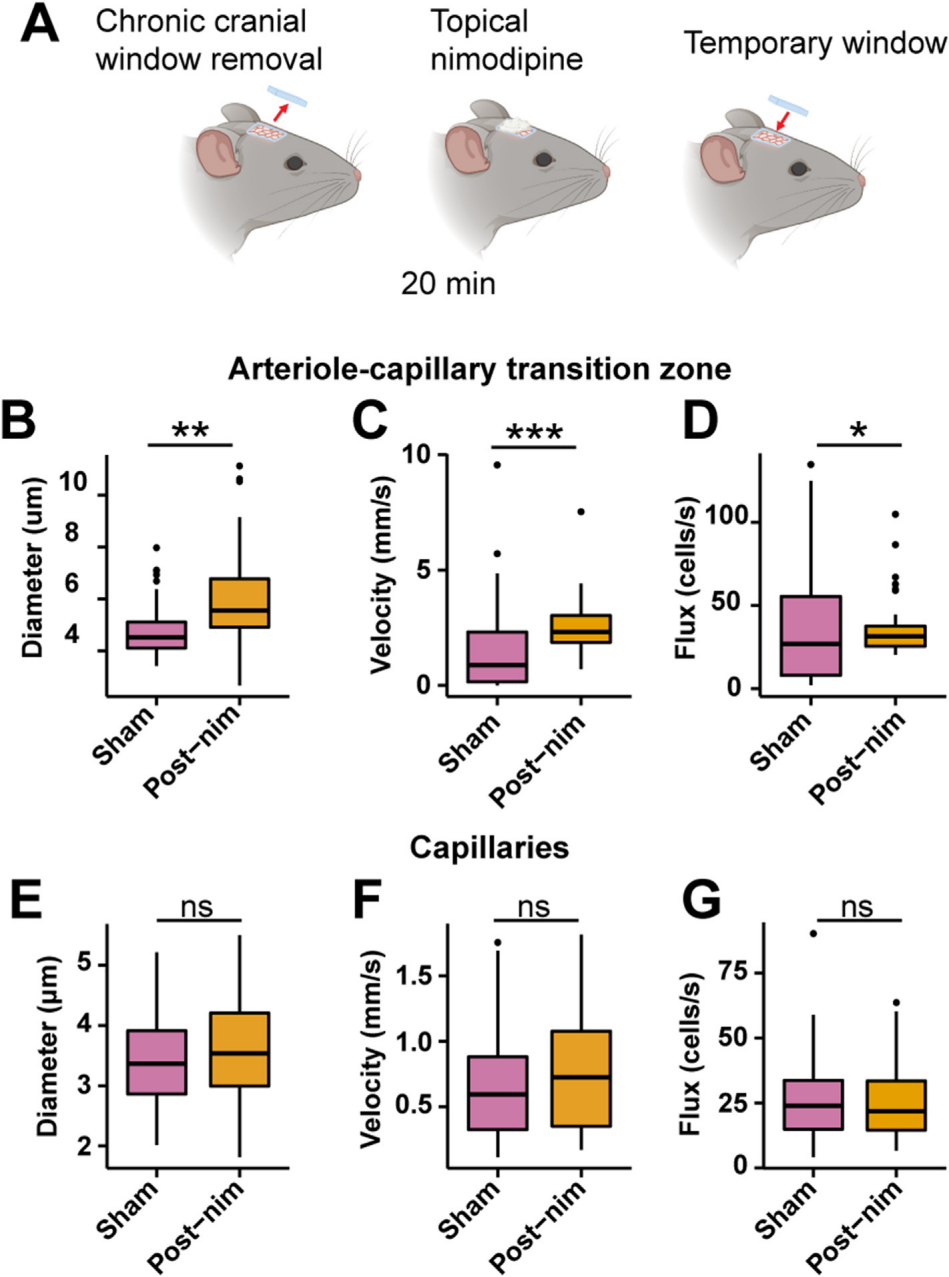
**Acute nimodipine has different effects on hemodynamics than systemic nimodipine. A)** Acute pharmacology experiment scheme where the window was removed and topical nimodipine was applied (10 ​μM) to the brain surface. **B)** Diameter, **C)** BC velocity, and **D)** BC flux of blood vessels from the transition zone covered by ensheathing pericytes. NSh ​= ​3 mice; nShV ​= ​22 vessels; *N*-nim ​= ​4 mice; n-nim V ​= ​38 vessels. **E)** Diameter, **F)** BC velocity, and **G)** BC flux of brain capillaries covered by thin-strand pericytes. NSh ​= ​3 mice; nShV ​= ​48 vessels; *N*-nim ​= ​4 mice; *n*-nimV ​= ​34 vessels. ∗P ​< ​0.05,∗∗P ​< ​0.01, ∗∗∗P ​< ​0.001.

We found that systemic nimodipine prevented the measurement of blood pressure with a non-invasive blood pressure tail-cuff system during the two-photon imaging (Supplementary Fig. 3A and B), likely due changes in tail volume [35] that could be hypotension-induced. This is a potential limitation of systemic nimodipine administration [41,42]. To determine if the effects caused by nimodipine i.p. injection were due to systemic changes rather than local cerebrovascular effects, we removed the cranial window, applied nimodipine (10 ​μM) directly to the surface of the brain, and repeated our calcium imaging and hemodynamic measurements (Fig. 7A). Topical nimodipine did not affect blood pressure (Supplementary Fig. 3C) but had similar effects as systemic nimodipine on Ca^2+^ signaling in ensheathing and thin-strand pericytes, causing a decrease in Ca^2+^ event amplitude and frequency compared to sham animals with the window removed but no nimodipine applied (Supplementary Fig. 4). Topical nimodipine also dilated vessels in the ACT, much like systemic nimodipine (Fig. 7B). However, this resulted in increased BC velocity and flux through the ACT (Fig. 7C and D), which is expected based on vascular resistance principles, but opposes the effects of systemic nimodipine (Fig. 3). Topical nimodipine did not affect capillary diameter or BC velocity and flux (Fig. 7E–G), suggesting that reduced BC hemodynamics with systemic nimodipine (Fig. 6) are dictated by intrinsic, upstream alterations in blood flow.

**Fig. 8:**
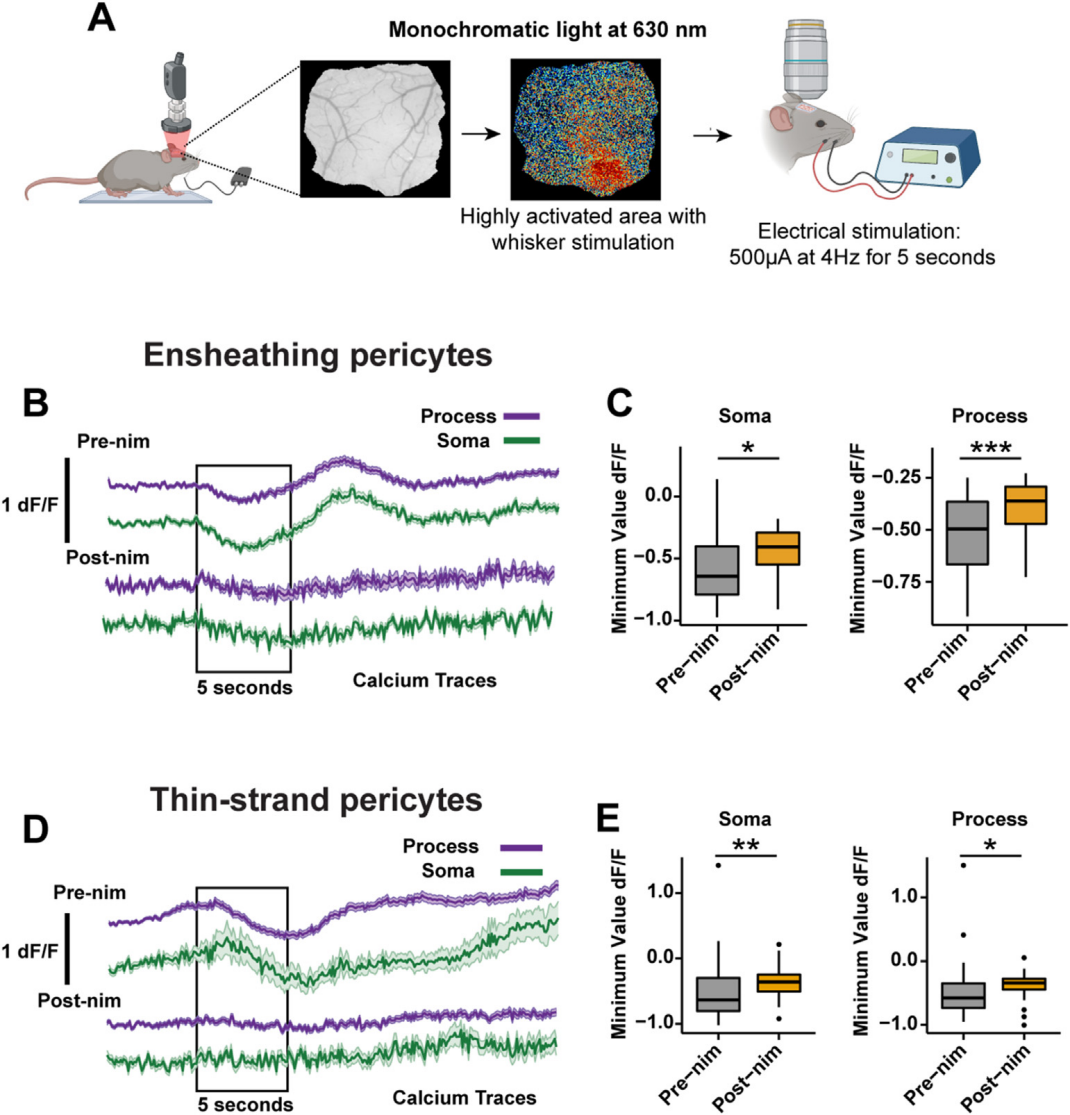
**Nimodipine attenuates pericyte Ca^2+^ behavior during neurovascular coupling. A)** Experimental set up of intrinsic optical imaging and electrical whisker stimulation for neurovascular coupling. The red area is an example from one mouse of a region high activated by whisker vibrations (average of 20 trials of stimulation). **B)** Ca^2+^ signaling traces of ensheathing pericytes during NVC. The black box represents the 5 ​s of electrical whisker stimulation. The traces show the mean value ​+ ​SEM over 25 s. **C)** Minimum value of Ca^2+^ drop in amplitude from ensheathing pericyte somata and processes during the stimulation period. n ​= ​33 pericytes in 5 mice. **D)** Ca^2+^ signaling traces of thin-strand pericytes during NVC. The black box represents the 5 s of electrical whisker stimulation. The traces show the mean value ​+SEM over the 25 s. **E)** Minimum value of the Ca^2+^ drop in amplitude from thin-strand pericyte somata and processes during the stimulation period. n ​= ​42 pericytes from 7 mice. ∗P ​< ​0.05, ∗∗∗P ​< ​0.001. Pre-nim ​= ​pre-nimodipine; Post-nim ​= ​post-nimodipine.

### Systemic nimodipine affects dilation during neurovascular coupling

**Fig. 9:**
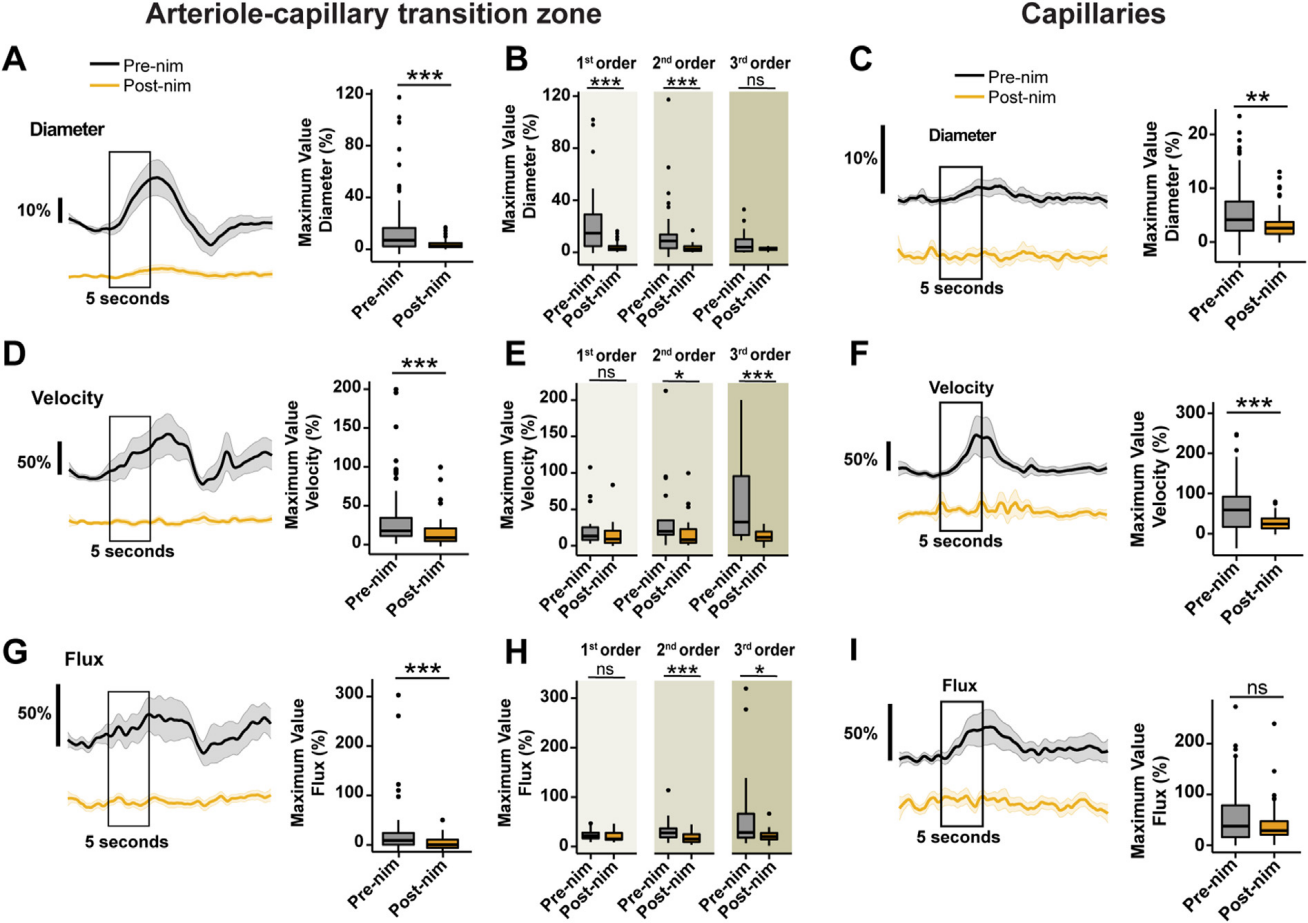
**Nimodipine attenuates hemodynamic responses during neurovascular coupling. A)** Left: Mean traces of the percentage change in diameter of vessels from the arteriole-capillary transition zone. Error bars are SEM and the black box represents the 5 ​s of electrical whisker stimulation. Right: Boxplots of maximum ACT vessel diameter during stimulation. n ​= ​65 vessels in 5 mice. **B)** Maximum diameter during stimulation in each branch of the ACT. b1 ​= ​16, b2 ​= ​21, b3 ​= ​19 vessels in 5 mice. **C)** Left: Traces of the percentage change in diameter in brain capillaries during electrical stimulation. Error bars are SEM and the black box represents the 5 ​s of electrical whisker stimulation. Right: Boxplots of maximum capillary diameter during stimulation. n ​= ​96 capillaries from 7 mice. **D)** Left: Traces of the percentage change in BC velocity in the ACT during electrical stimulation. Right: Boxplots of the maximum BC velocity during stimulation. **E)** Maximum velocity during stimulation in each branch of the ACT. **F)** Left: Traces of the percentage change in BC velocity in brain capillaries during electrical stimulation. Right: Boxplots of maximum capillary BC velocity during stimulation. **G)** Left: Traces of the percentage change in BC velocity in the ACT during electrical stimulation. Right: Boxplots of the maximum BC velocity during stimulation. **H)** Maximum velocity during stimulation in each branch of the ACT. **I)** Left: Traces of the percentage change in BC velocity in brain capillaries during electrical stimulation. Right: Boxplots of maximum capillary BC velocity during stimulation. ∗∗∗P ​< ​0.001. Pre-nim ​= ​pre-nimodipine; Post-nim ​= ​post-nimodipine.

NVC is a fundamental process where neuronal activity triggers vasodilation to ensure that active brain regions receive more blood to meet increased energy demand. Since nimodipine is a vasodilator, we wondered if it would change the response of pericytes to NVC cues. First, we mapped the whisker barrel cortex of mice by intrinsic optical imaging to identify regions within the cranial window that responded to whisker vibrations (Fig. 8A). Then, we selected ensheathing or thin-strand pericytes in this active area during two-photon imaging and we applied a mild electrical stimulus (500 ​μA ​at 4 ​Hz for 5 s) to the whisker pad to induce NVC. We calculated the change in pericyte Ca^2+^ or blood flow during stimulation relative to the pre-stimulus baseline period (5 s). Typical NVC responses were observed during basal conditions where the stimulus caused a drop in Ca^2+^ in ensheathing pericyte and thin-strand pericyte somata and processes (Fig. 8B–E), which correlated with a large dilation of vessels in the ACT zone, particularly in the 1st branch (Fig. 9A), and capillary dilation (∼3 ​%, Fig. 9B, pre-nimodipine session). Following systemic treatment with nimodipine, the pericyte Ca^2+^ decrease (Fig. 8B–E) and vessel dilation during the stimulus where abolished throughout the vascular network (Fig. 9A–C) with the strongest effects on dilation in the 1st and 2nd branches of the ACT (Fig. 9B). Vessel dilation during NVC leads to increased BC velocity and flux throughout the ACT and capillary network, and systemic nimodipine prevented this change in blood flow during stimulation (Fig. 9D–I). Notably, the change in velocity or flux during NVC was greatest in the 2nd and 3rd branches of the ACT, which is also where nimodipine caused the greatest attenuation (Fig. 9E–H). This likely occurs because strong dilation of the 1st branch drives faster velocities in downstream branches and if nimodipine has greater effects on the 1st branch it would have a larger impact on velocity in the downstream network.

## Discussion

Even though nimodipine is given to patients after SAH, we know little about how it affects brain microvascular hemodynamics and pericyte contractility. Recent studies in a mouse SAH model showed that pial artery vasospasms are apparent within a few hours of SAH induction [5,43] and these vasospasms are alleviated by nimodipine (10 ​mg/kg) [5]. Importantly, pericyte constriction was not observed in this model [43], suggesting these cells are not prone to vasospasm in SAH. Nevertheless, since L-type VGCCs contribute to pericyte Ca^2+^ events [13,17,27,44] and contraction of vessels in the ACT [16,28], nimodipine has the potential to impact pericyte physiology and alter hemodynamics throughout the microvascular network. Here, we show that systemic nimodipine decreases calcium signaling in all pericyte types across the healthy cerebrovascular network, and it causes basal vasodilation of ensheathing pericytes and reduced vasomotion. Vasomotion is known to drive paravascular clearance [39] and induce periodical fluctuations in blood flow (i.e. “flowmotion”) that facilitates downstream tissue dialysis and homeostasis, particularly during times of metabolic stress [38,40]. As such, nimodipine may disrupt this important vascular homeostatic process by reducing vasomotion, which may also partly explain why systemic nimodipine also decreased BC velocity throughout the vascular network (from ACT to capillaries).

Nimodipine is known to preferentially dilate small cerebrovascular arteries (<70 ​μm), which is attributed to the VGCC subunit composition in these vessels [1]. Therefore, it is interesting that we observed a gradient of nimodipine-induced vasodilation (increased mean basal diameter) with the strongest response in the 1st branch to no response in the 3rd branch or capillaries. The 1st branch is known to have the most robust response to vasodilatory and vasoconstrictor stimuli [19] and the fastest onset of dilation during NVC [18]. No basal dilation in the 3rd branch or capillaries by nimodipine may reflect a decrease in VGCC activity [16] or a decreasing gradient of αSMA expression by pericytes [12,19,20,22,45]. Overall, our results suggest VGCC are highly active in ensheathing pericytes within the ACT most proximal to arteriole and contribute to the vascular dynamics throughout this region.

We also show for the first time *in vivo* that nimodipine decreases capillary pericyte Ca^2+^ signal amplitude and frequency in somata and processes. This contrasts *ex vivo* brain slice studies with dihydropyridines where nimodipine caused a mild Ca^2+^ frequency decrease limited to capillary pericyte somata, while nifedipine had no effect on Ca^2+^ frequency [17,27]. Nevertheless, while we observed a reduction in capillary pericyte Ca^2+^ signaling with systemic or acutely applied nimodipine, there was no change in capillary diameter. This suggests that VGCC do not contribute to resting capillary tone.

We also found that systemic nimodipine decreased BC velocity in all vessel types from the ACT zone (Fig. 3F) to higher order capillaries (Fig. 6D). However, there was no difference BC flux in the ACT vessels (Fig. 3, Fig. 4F), even though there was a reduction in BC flux in the capillaries (Fig. 6E). This can be explained by the velocity-flow relationships assuming laminar flow and the following formula Flow ​= ​Velocity ∗ Vessel Radius^2^ [46]. When vessel diameter is constant, velocity alterations induce proportional changes in flow (i.e. flux). However, if the diameter of a blood vessel increases including in downstream vessels as we observed through the ACT zone, the blood velocity would decrease, but the flux would remain unchanged. At the capillary level, since there was no capillary dilation, when the velocity decreases in capillaries (particularly because of upstream reductions in velocity) then the flux decreases as well, complying with the velocity-flow relationship. Overall, these nimodipine effects on cerebrovascular flow were likely due to systemic influences on other blood vessels in the body and potentially reduced blood pressure (Supplementary Fig. 3), since we did not observe these changes when nimodipine was applied locally (Fig. 7).

It should be noted that nimodipine can also block VGCC on other brain cells, such as neurons, which could affect signaling within the neurovascular unit. But, a higher dose of nimodipine (10 ​mg/kg) than what we used in this study does not reduce neuronal potentials evoked by sensory whisker stimulation [47], so we would expect neuronal responses to the electrical stimulation to remain intact and evoke NVC. However, we found that electrical stimulation did not evoke vessel dilation or changes in BC velocity/flux in the ACT zone or capillaries in the presence of nimodipine. This could happen for two reasons: 1) Given that nimodipine causes dilation of the first and second branches in the ACT (Fig. 4), it is possible that enough oxygen and nutrients are delivered to the downstream tissue to meet the energy demands of active neurons. Therefore, further dilation from NVC may not be necessary. 2) Alternatively, dilation during NVC may not be able to overcome the vessel relaxation induced by nimodipine and blockade of VGCC may prevent VGCC deactivation on pericytes during NVC. When considering capillaries, we observed that nimodipine (systemic or acutely applied) dilates vessels in the ACT (Fig. 3) without changing downstream resting capillary diameter (Fig. 6), suggesting that capillaries do not passively dilate when upstream vessels are dilated. Notably, a transient capillary dilation during sensory stimulation did not occur in the presence of nimodipine (Fig. 9), even though there was no change in resting capillary diameter. This reinforces the notion that resting hemodynamic changes by nimodipine are enough to meet the energy demands of the tissue during sensory stimulation. However, it may also suggest that a transient drop in thin-strand capillary pericyte Ca^2+^ activity is required to permit capillary dilation during NVC and when this is not present, as is the case following nimodipine treatment (Fig. 8), capillary dilation is prevented.

Our study has several limitations. First, we used ketamine/xylazine anesthesia, which has been used in other studies of pericyte Ca^2+^ and blood flow [18,48] and permitted repetitive dosing of the same animals in different pre-nimodipine and post-nimodipine imaging sessions. While we observed robust and consistent neurovascular coupling responses in all animals that were in line with other studies [11,18,48,49], we can not rule out the possibility that the anesthetic affected some blood vessels more than others. Future studies in awake animals would clarify the influence of anesthetics in this case. Second, we measured blood pressure by the tail-cuff method, which is challenging in mice because it is heavily influenced by tail temperature, vascular tone and blood volume of the peripheral tail arteries [35]. Also, tail cuff measurements in mice do not always correlate with central blood pressure, particularly if vasoactive substances change the volume of the tail arteries [35,[50], [51], [52]]. Since nimodipine is a vasoactive substance, we suspect that it reduced blood pressure in our animals, but it may have changed tail volume to a point where blood pressure could not be detected (Supplementary Fig. 3). We can not say for certain due to the limitations of this approach. Therefore, future experiments with a more accurate method to measure arterial pressure, such as a fluid filled catheter or implanted telemetry system, are necessary to determine if the concentration of nimodipine used in this study (1 ​mg/kg) induces a drop in central blood pressure. Third, topical drug application on the surface of the brain has been used for study of hemodynamics or even animal behavior [32,[53], [54], [55]], but it relies on the diffusion of the drug into the tissue. We observed a strong reduction in pericyte Ca^2+^ when nimodipine was applied topically (Supplementary Fig. 4), suggesting that nimodipine reached the vessels where we made our measurements, but it is possible that the nimodipine concentration was different at deeper vessels. Lastly, we included both males and females in our study, but our sample size was not sufficiently powered for sex comparisons.

Nimodipine is typically administered to patients orally (60 ​mg every 4 ​h) or intravenously (15 ​ ​μg/kg/hour) in the days following SAH [1,2]. The dose of nimodipine used in our study (1 ​mg/kg, i.p.) is lower than the dose from other animal studies (10 ​mg/kg i.p [5,47].), but nevertheless, shows clear impacts on pericyte Ca^2+^ activity and BC velocity. These reductions in BC velocity are due to the systemic action of nimodipine, since we found acute application topically on the surface of the brain increased BC velocity throughout the dilated vessels of the ACT. Therefore, systemic nimodipine may affect pericyte physiology and cause a redistribution of CBF when it is used clinically in SAH patients. Further study of SAH and the effects of nimodipine on pericytes and CBF throughout the cerebrovascular network will be an interesting future direction.

## Author contributions

J.M.R. and J.S. designed the study. J.M.R., S.K. N.A., M.K., M.S., J.D.R., M.R., D.K. collected the data. J.M.R., F.O., and J.S. analyzed data. J.M.R. and J.S. wrote the paper. C.G. and B.W provided resources and revised the manuscript.

## Declaration of competing interest

The authors declare that they have no known competing financial interests or personal relationships that could have appeared to influence the work reported in this paper.
