# Supplementary material for: Systemic nimodipine affects pericyte calcium signaling, resting hemodynamics and neurovascular coupling in healthy mouse brain
Source: Neurotherapeutics. 2025 May 21;22(5):e00614. doi: 10.1016/j.neurot.2025.e00614 (PMC12491807; doi:10.1016/j.neurot.2025.e00614)
Supplement: Multimedia component 2 [file mmc2.docx]

**Supplementary Information**

**Systemic nimodipine affects pericyte calcium signaling, resting hemodynamics and neurovascular coupling in healthy mouse brain.**

Jessica Meza-Resillas^1,2^, Finnegan O’Hara^1^, Syed Kaushik^1^, Michael Stobart^1^, Noushin Ahmadpour^1^, Meher Kantroo^1^, John Del Rosario^1^, Megan C. Rodriguez^1^, Dmytro Koval^1^, Chaim Glück^3^, Bruno Weber^3^, Jillian Stobart^1,2^* –

1. College of Pharmacy, University of Manitoba, Winnipeg, Manitoba, Canada
2. Centre on Aging, University of Manitoba, Winnipeg, Manitoba, Canada
3. Institute of Pharmacology and Toxicology, University of Zurich, Zurich, Switzerland

*Corresponding author: Jillian Stobart.

**Email:**  [jillian.stobart@umanitoba.ca](mailto:jillian.stobart@umanitoba.ca)

**
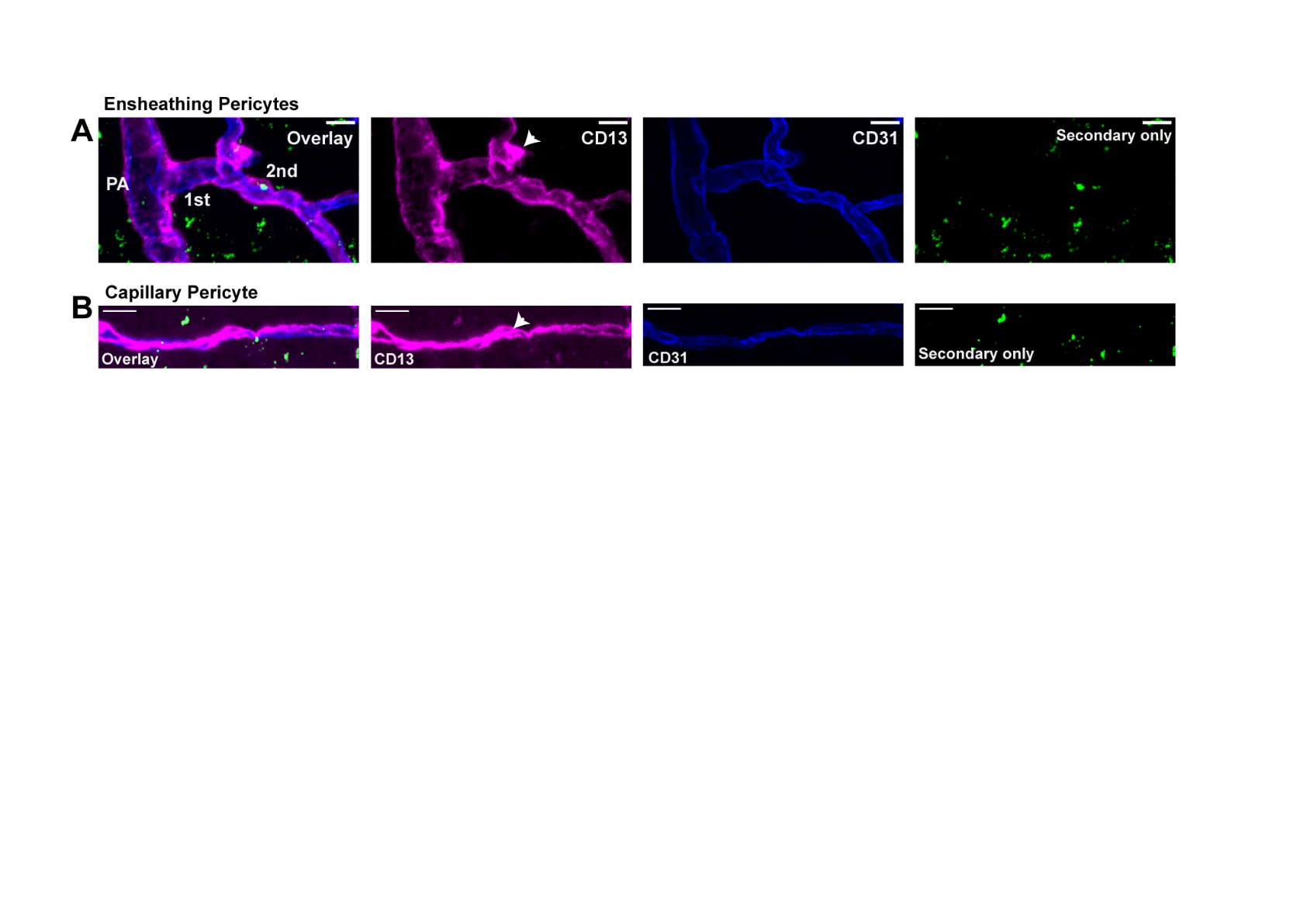
**

**Supplementary Figure 1. Cav1.2 control immunostaining where primary antibody for Cav1.2 was omitted.** Vascular secondary antibody staining was not observed in ensheathing pericytes (A) or capillary pericytes (B) labelled with anti-CD13 (magenta) near endothelial cells (anti-CD31, blue). Arrowheads indicate pericyte somata. All scale bars = 10 µm.


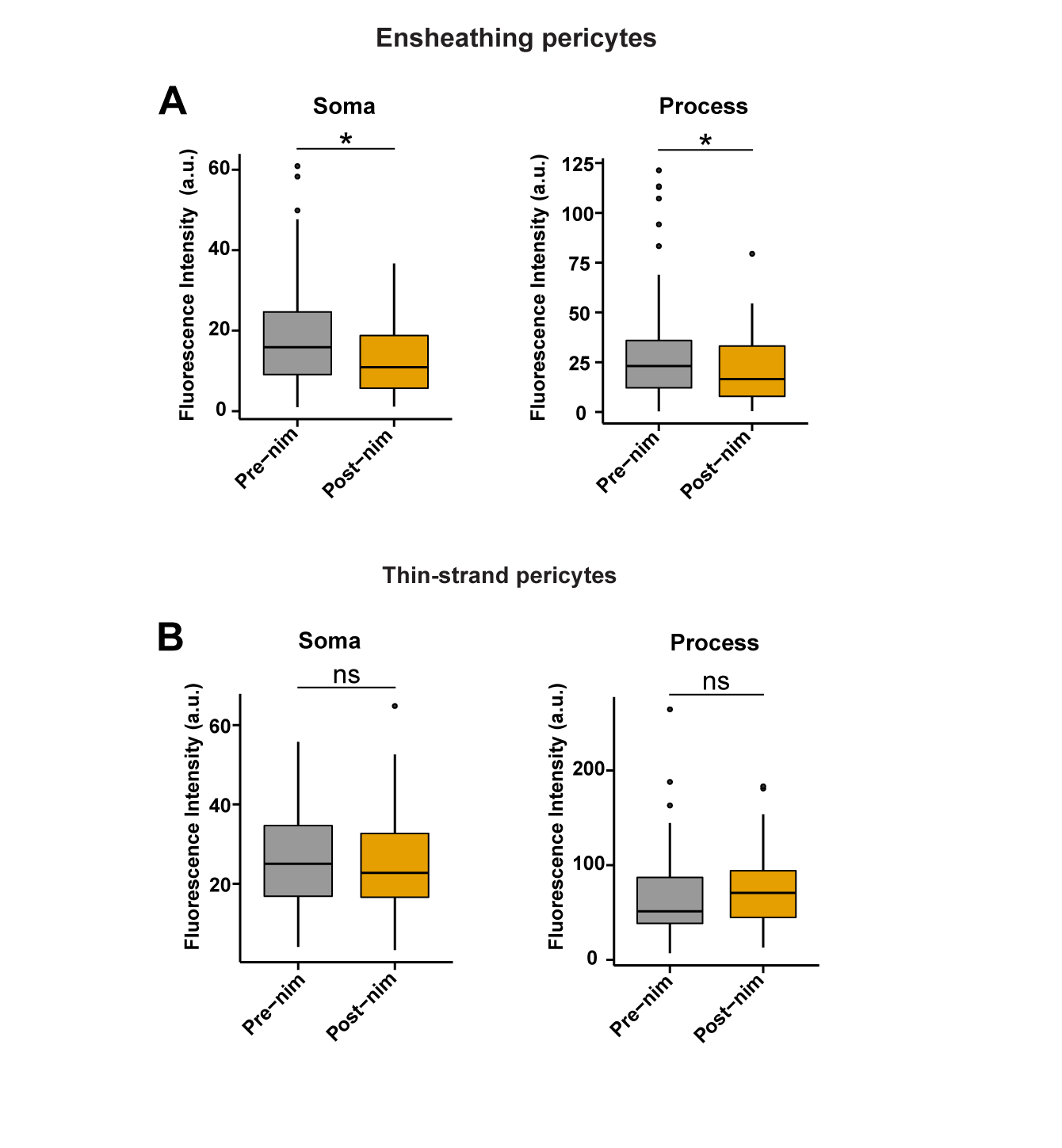


**Supplementary Figure 2. The effects of nimodipine on basal pericyte Ca^2+^ intensity (F_0_).** Box plots of the basal Ca^2+^ fluorescence intensity (a.u. = arbitrary units) of **A)** ensheathing pericytes and **B)** capillary pericytes somata and processes in pre-nimodipine and post-nimodipine conditions. n= 43 ensheathing pericytes from 7 mice and 44 capillary pericytes from 7 mice. * P<0.05; ns= no significant difference.

**
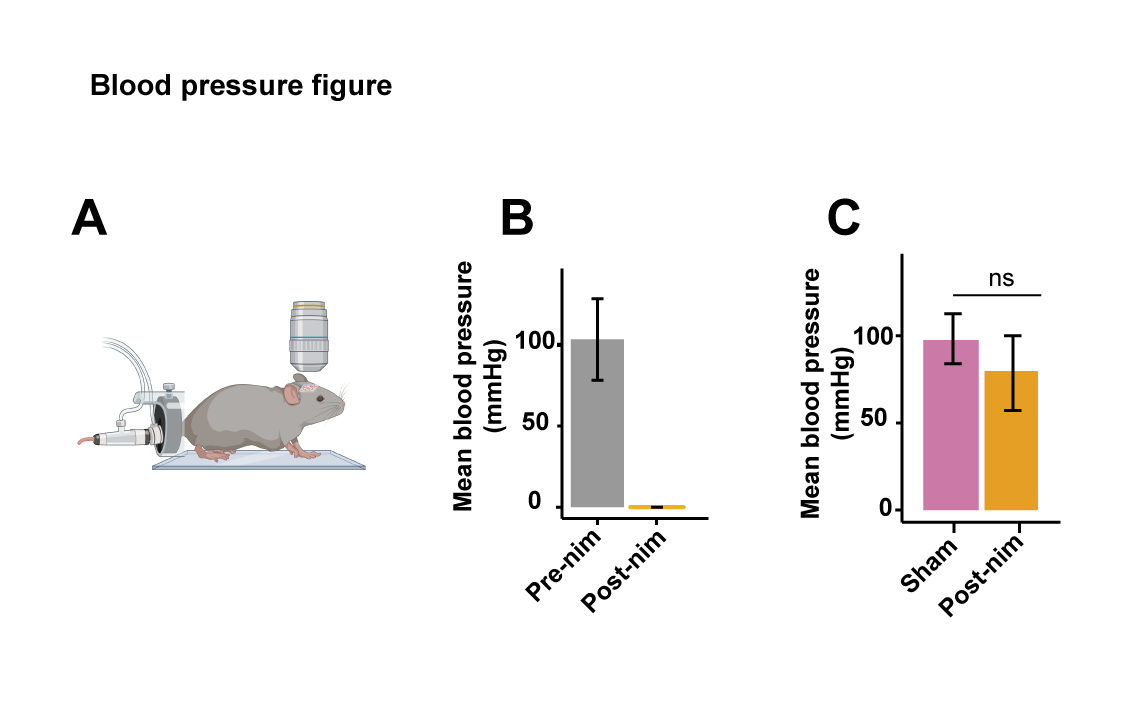
Supplementary Figure 3. Blood pressure measurements. A)** The experimental set up of CODA monitor non-invasive blood pressure system. **B)** Mean blood pressure measured in repeated pharmacology experiments of mice in pre-nimodipine and post-nimodipine applied via i.p. CODA system failed to report blood pressure after nimodipine was applied, suggesting that blood pressure decreased below the detection of the system or that the tail volume was altered significantly altered preventing accurate measurements with the system. N=11 mice. **C)** Mean blood pressure of mice in sham and post-nimodipine acute pharmacology experiments. Sham=6 mice; Post-nim=9 mice. ns= no significant difference.

No values detected

**
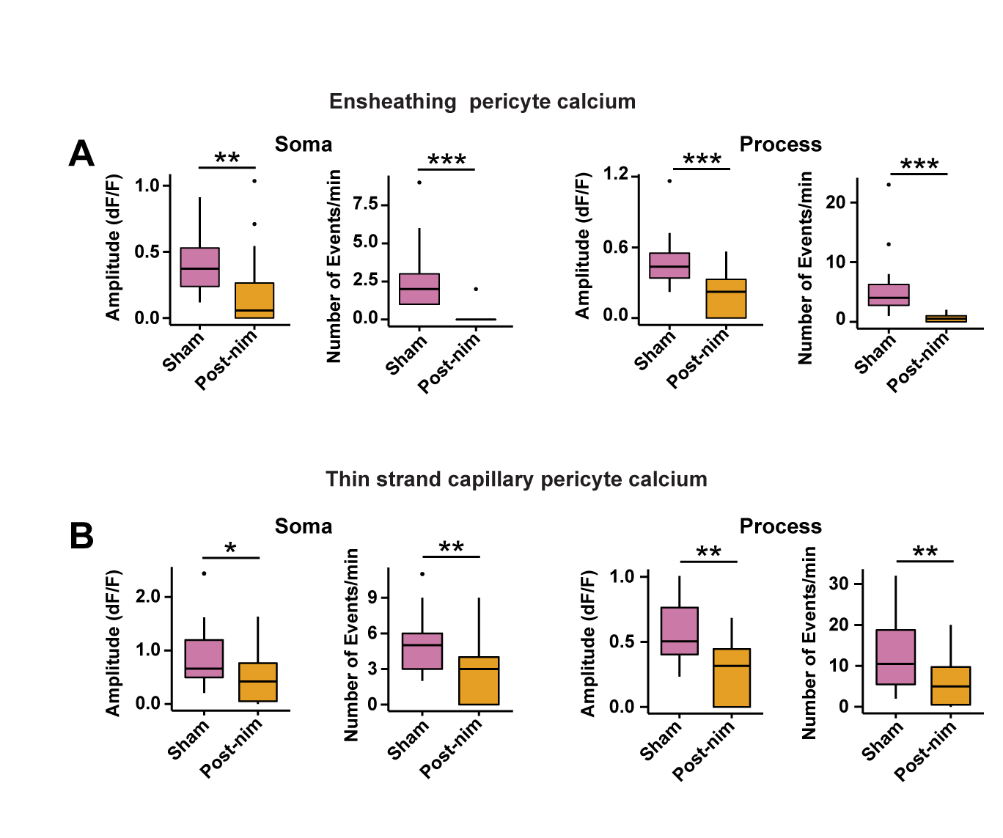
Supplementary Figure 4. Acute nimodipine application reduces pericyte calcium activity. A)** Calcium signaling properties (amplitude and frequency) of ensheathing pericyte soma and process. Sham= 3 mice; Sham= 28 pericytes; Post-nim= 4 mice; Post -nim= 34 pericytes. B**)** Calcium signaling properties (amplitude and frequency) of thin-strand pericyte soma and process. Sham= 3 mice; Sham= 21 pericytes; Post -nim= 3 mice; Post -nim= 26 pericytes.
